# The Tool for Evaluating Media Portrayals of Suicide (TEMPOS): Development and Application of a Novel Rating Scale to Reduce Suicide Contagion

**DOI:** 10.3390/ijerph19052994

**Published:** 2022-03-04

**Authors:** Chloe Chang Sorensen, Mego Lien, Vicki Harrison, John J. Donoghue, Jeevanjot Singh Kapur, Song Hi Kim, Nhi Thi Tran, Shashank V. Joshi, Sita G. Patel

**Affiliations:** 1 Depression Clinical & Research Program, Massachusetts General Hospital, Boston, MA 02114, USA; 2 Suicide Prevention Program, County of Santa Clara Behavioral Health Services Department, San Jose, CA 95128, USA; mego.lien@hhs.sccgov.org (M.L.); john.donoghue@hhs.sccgov.org (J.J.D.); 3 Department of Psychiatry & Behavioral Sciences, Stanford University, Stanford, CA 94305, USA; jjkapur@stanford.edu (J.S.K.); songhkim@stanford.edu (S.H.K.); svjoshi@stanford.edu (S.V.J.); 4 Center for Care Innovations, Oakland, CA 94612, USA; nhi@careinnovations.org; 5 Department of Psychology, Palo Alto University, Palo Alto, CA 94304, USA; spatel@paloaltou.edu

**Keywords:** suicide, suicide prevention, safe messaging, imitative suicide, suicide contagion, media-influenced harm, media reporting, werther effect, papageno effect, program evaluation

## Abstract

Research suggests that media adherence to suicide reporting recommendations in the aftermath of a highly publicized suicide event can help reduce the risk of imitative behavior, yet there exists no standardized tool for assessing adherence to these standards. The Tool for Evaluating Media Portrayals of Suicide (TEMPOS) allows media professionals, researchers, and suicide prevention experts to assess adherence to the recommendations with a user-friendly, standardized rating scale. An interdisciplinary team of raters constructed operational definitions for three levels of adherence to each of the reporting recommendations and piloted the scale on a sample of articles to assess reliability and clarify scale definitions. TEMPOS was then used to evaluate 220 news articles published during a high-risk period following the suicide deaths of two public figures. Post-hoc analyses of the results demonstrated how data produced by TEMPOS can be used to inform research and public health efforts, and inter-rater reliability analyses revealed substantial agreement across raters and criteria. A novel, wide-reaching, and practical approach to suicide prevention, TEMPOS allows researchers, suicide prevention professionals, and media professionals to study how adherence varies across contexts and can be used to guide future efforts to decrease the risk of media-induced suicide contagion.

## 1. Introduction

Suicide is one of the leading causes of death worldwide, with over 800,000 people dying by suicide annually, more than war, malaria, or breast cancer [1]. Media representations of suicide can influence the contagion of suicidal behavior, particularly in vulnerable populations. Rates of self-harm and suicide attempts have been increasing in recent years [2,3], and a growing body of research has established a link between self-harm and increased media use [4,5,6]. Suicide mortality tends to increase following highly publicized suicide events, a phenomenon known as the Werther Effect [7]. The association between media reporting and suicide contagion is well established as a globally reaching public health concern, documented in over 150 empirical studies and systematic reviews from around the world [8,9,10,11,12]. Newspaper coverage of suicide has been found to be significantly associated with the initiation of suicide clusters [13], and a substantial number of suicide attempt survivors report being affected by a media story about suicide [14,15]. Increases in suicide rates following a highly publicized suicide event tend to be proportionate to the volume, duration, and prominence of the coverage [16], and are greater when the majority of the coverage is sensationalistic or includes details about the suicide method [17,18]. For example, in the three months following the highly publicized suicide of American comedian Robin Williams in 2014, there were 16% more suicides than expected; moreover, the greatest increases were seen in deaths by asphyxiation (the method used by Williams) and in males over 30, suggesting an imitative effect [19].

Despite these findings, media can also play an important role in suicide prevention [18,20]. When media outlets minimize the inclusion of certain kinds of harmful information (e.g., information about how the suicide was completed), the risk of imitative behavior decreases [18,20]. For example, a campaign by suicide prevention experts in Austria to implement guidelines for reporting on railway suicides in Vienna in the 1980’s led to a reduction in the volume of coverage and, in turn, an 84% reduction in suicides [21]. More recent research demonstrates that media reports that portray suicide as a preventable outcome and disseminate resources and information about suicide prevention can help decrease suicide rates, a phenomenon known as the Papageno Effect [20,22].

In order to help media professionals decrease the risk of imitative suicide and instead promote a Papageno Effect, the World Health Organization published a set of recommendations for safely reporting on suicide [23]. These recommendations—which draw upon decades of research on suicide contagion and were developed with input from leading experts in the fields of suicide prevention, journalism, and public health—are continually expanded and adapted to reflect current empirical understanding. In addition to the WHO recommendations, suicide prevention professionals around the world have created and disseminated similar sets of recommendations, such as the Recommendations for Reporting on Suicide, developed in the United States [24], the Mindset guidelines from Canada [25], and the Mindframe guidelines from Australia [26]. Despite the existence of many different sets of recommendations, their content is consistent: Media professionals are advised to avoid sensationalizing or glamorizing the person who died, to avoid including explicit details about the death or suicide method, and to include prevention resources for those who may be struggling and at risk of suicide. Adherence to such guidelines is associated with a reduction in suicide rates [27], decreased use of highly lethal suicide methods [28], and increased utilization of support resources [20].

Adherence to these guidelines is especially vital during surges of suicide-related coverage, such as when a high-profile figure dies by suicide. Famed American fashion designer Kate Spade died by suicide on 5 June 2018, leading to a spike in suicide-related news coverage in the United States. Celebrity chef and TV personality Anthony Bourdain took his own life just three days later, on 8 June 2018. Celebrity suicides that occur in such close succession are exceedingly rare but provide a valuable opportunity to study how media outlets cover suicide. However, there currently exists no standardized method for measuring adherence levels, making it difficult to compare results across studies or understand how adherence varies across contexts.

Many studies examining adherence use a binary rating metric, noting the presence or absence of each recommended reporting practice [29,30,31]. Although simple to use, binary rating systems fail to account for the fact that degrees of adherence to a particular reporting recommendation may have differential impacts on the audience. For example, a newspaper article that provides graphic details about a suicide method may be significantly more harmful than an article that mentions the method in passing; however, under a binary rating system, these two articles would both be coded the same way. In order to capture more nuance in reporting, some researchers have utilized traditional content analysis methods [13,17,32,33], while others have developed their own rating methods tailored specifically to their research aims [34,35]. Although these more complex approaches succeed in capturing more nuance, they tend to be difficult and time-consuming to execute. Consequently, they are unlikely to be widely adopted by suicide prevention programs or media professionals in need of evaluation and monitoring tools.

The lack of a standardized, user-friendly rating system also poses a challenge to suicide prevention programs aiming to monitor and evaluate their progress in working with the media to increase adherence to the reporting recommendations. For example, in response to the clusters of youth suicides in 2009 and 2014, the Suicide Prevention Program of Santa Clara County in Northern California has been working with local media to improve suicide-related reporting since 2011. A 2016 study conducted by the Centers for Disease Control (CDC) in response to these clusters found that among the 246 media reports analyzed, only 17% included any sort of suicide prevention resource. On average, each media report contained 4.3 potentially harmful characteristics, compared to an average of only 0.5 protective characteristics [36]. After the study ended, however, program staff had no standardized method to assess the progress of their work with the media over time, to identify targeted areas for further improvement, or to provide quantified feedback to media partners about their adherence. Program leadership identified the need for the development of a new standardized assessment tool that would allow suicide prevention programs to track changes in media adherence over time and identify targeted areas for improvement.

To address these gaps, the County of Santa Clara’s Suicide Prevention Program and the Stanford Department of Psychiatry and Behavioral Sciences collaborated to develop the Tool for Evaluating Media Portrayals of Suicide (TEMPOS). The primary aim of this work is to develop a novel, user-friendly, and non-binary rating tool that can be used by members of the media (i.e., journalists, editors), suicide prevention professionals, and researchers. The second aim is to illustrate how TEMPOS may be used to monitor and evaluate media coverage. The present study describes the development of TEMPOS and its subsequent application to a dataset of 220 suicide-related news articles collected during a surge of suicide-related coverage. Through the process of applying the scale, tool characteristics and reliability were further explored.

## 2. Materials and Methods

### 2.1. Scale Development

TEMPOS was developed by an interdisciplinary team of researchers with backgrounds in psychology, psychiatry, public health, media, and community mental health. TEMPOS consists of ten criteria (Table 1), which were derived from the American version of the suicide reporting recommendations, the Recommendations for Reporting on Suicide [19]. Rather than utilizing a binary scoring system (adherence/non-adherence), TEMPOS utilizes a three-point rating scale to capture more complexity without being onerous. When using TEMPOS, a rating of 2 indicates full adherence to the guideline, a rating of 1 indicates partial adherence, and a rating of 0 indicates non-adherence. In order to delineate what qualifies as full, partial, or non-adherence, operational definitions were constructed for each rating level (three for each of the 10 criteria, and 30 definitions in total). Wherever possible, definitions were constructed using language drawn directly from reportingonsuicide.org (accessed on 28 January 2022), in order to maximize alignment with the recommendations.

**Figure 1:**
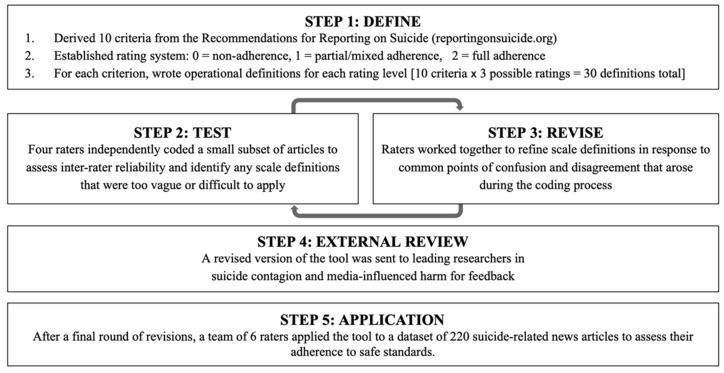
TEMPOS Development Process.

The interdisciplinary TEMPOS team followed a four-step scale development process (Figure 1). First, following initial construction of the 30 rating choices, the team discussed each operational definition and revised any wording that was deemed unclear or ambiguous. Second, the 3-point rating system was pilot-tested with a subsample of 5 suicide-related articles drawn from a larger dataset of 220. Four raters independently rated these articles in order to assess inter-rater reliability and identify any scale definitions that were too vague or difficult to apply. Third, the team reviewed the ratings and worked together to refine the scale definitions in response to common points of confusion and disagreement that arose during the pilot coding process.

The fourth step in scale development intended to strengthen the validity of the scale through an external review process with experts on suicide contagion and media-influenced harm. We invited five external reviewers, each an expert in suicide contagion and media-influenced harm, to provide feedback and critique of TEMPOS. Each external reviewer was sent a draft of the scale and asked to provide comments on the structure of the scale, as well as the wording and validity of the constructs. Based on the feedback from these external reviewers, the team revised the scale and completed one final round of test coding on a subsample of 10 articles in order to assess inter-rater reliability prior to applying the scale to the full dataset of 220 articles.

### 2.2. Suicide News Media Dataset

The suicide deaths of Kate Spade and Anthony Bourdain in early June of 2018 triggered a surge in suicide-related coverage, providing a natural opportunity to study how regional and national news outlets cover suicide. Over the course of a month, the County of Santa Clara’s Suicide Prevention Program compiled a dataset of suicide-related news articles published in the United States. Articles were obtained using Google Alerts and manual searches of the keywords “suicide”, “suicide prevention”, “mental health”, “mental illness”, and “self-harm”. Letters to the editor, articles from publications focused on gossip (e.g., TMZ), non-English articles, obituaries, and articles covering murder-suicides were excluded.

### 2.3. Application of Scale

To illustrate how TEMPOS may be used to monitor and evaluate media coverage, the first author applied all ten TEMPOS criteria to each of the 220 articles in the dataset. In order to assess the inter-rater reliability of the scale, each article was also independently rated by one of five secondary raters.

Following the completion of the coding process, raters met to discuss and resolve discrepancies between the two sets of ratings in order to examine the inter-rater reliability of TEMPOS when applied to a large sample of media and produce a final set of ratings. In addition to rating each article for adherence to each of the ten criteria, researchers also calculated an overall TEMPOS score by dividing the total number of points scored by the total number of points possible. If any criteria were rated as “not applicable”, the total number of points possible was adjusted (20 total minus 2 points for each criterion that was rated “not applicable”). For ease of interpretation, scores were converted to percentages, with 0% indicating total non-adherence to the reporting recommendations, and 100% indicating full adherence.

## 3. Results

### 3.1. Characteristics of the Dataset

**Figure 2:**
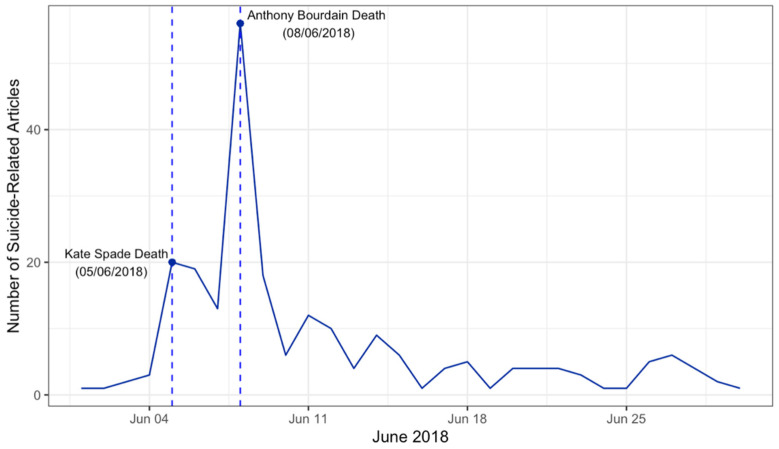
Surge in Articles Following Two High Profile Deaths by Suicide.

In total, 226 articles were collected from several media outlets covering national and local Bay Area news, including broadcast networks, online magazines and newspapers, and blogs that had readership of at least 1000 people. By the time the scale was fully developed, six articles from the dataset were no longer available, leaving a total of 220 articles. As expected, there was a surge in suicide-related coverage immediately following the death of Kate Spade, and coverage peaked three days later following the death of Anthony Bourdain (Figure 2). Article characteristics are presented in Table 2.

### 3.2. Inter-Rater Reliability

Inter-rater reliability was calculated by identifying the number of agreements between the two sets of ratings for each article and calculating the overall percentage of agreement. Across all raters and criteria, pure inter-rater agreement was 81.31%. To adjust for chance agreements, we calculated Cohen’s Kappa (κ) for each criterion [37]. Since the κ statistic depends on marginal values to calculate chance agreement, low prevalence of a variable can produce lower κ values. Accordingly, reporting characteristics that displayed low variability–for example, very few articles in our dataset contained content that glamorized suicide–displayed significantly lower κ values. Therefore, in addition to Cohen’s κ, percentage agreement is also presented for each criterion (Table 3). The average κ value across all criteria was 0.62; a κ value between 0.6 and 0.8 is generally understood to indicate substantial agreement among raters [37].

### 3.3. Analysis of TEMPOS Scores

**Figure 3:**
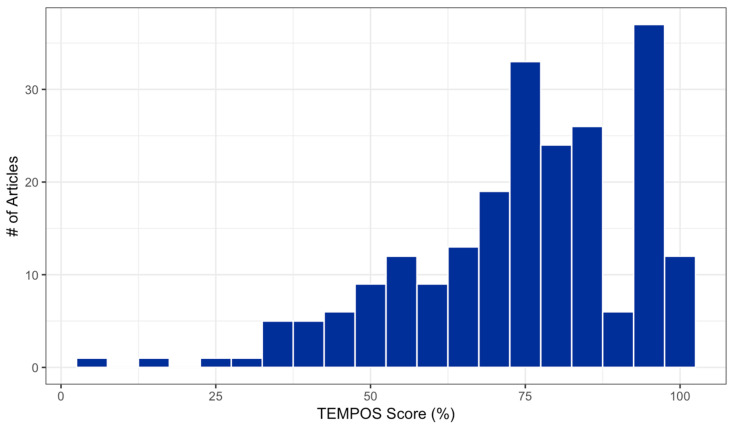
Distribution of TEMPOS scores.

We performed a series of exploratory analyses to understand overall levels of adherence, as well as how adherence levels varied between publications, across criteria, and over time. Overall TEMPOS percentage scores ranged from 5% to 100% (*M* = 74.7%, *MDN* = 75.0%, and *SD =* 18.2%). The distribution of overall scores was negatively skewed, as illustrated in Figure 3.

**Figure 4:**
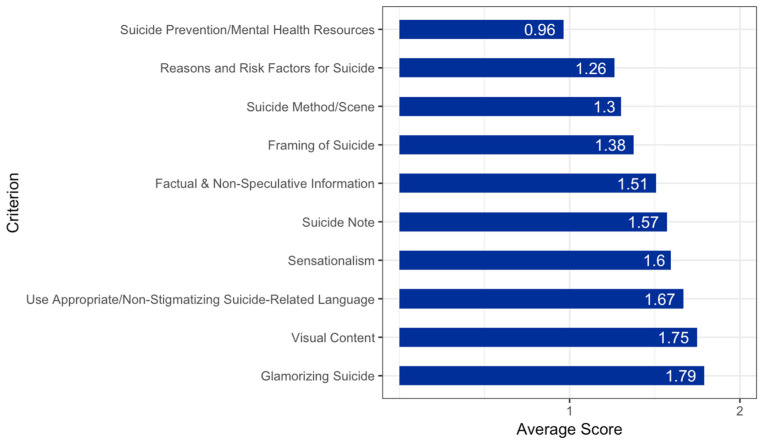
Average Scores for Evaluation Criteria.

Adherence levels varied significantly by criterion (Figure 4). The criterion that displayed the lowest mean levels of adherence was “suicide prevention and mental health resources” (*M* = 0.96, *SE* = 0.05), which aligns with past findings that media reports of suicide often fail to provide information and resources that could help those who may be struggling [29,30,38]. The criterion that displayed the highest mean levels of adherence was “glamorization of suicide” (*M* = 1.79, *SE* = 0.03), suggesting that very few media outlets portray suicide in a positive manner.

**Figure 5:**
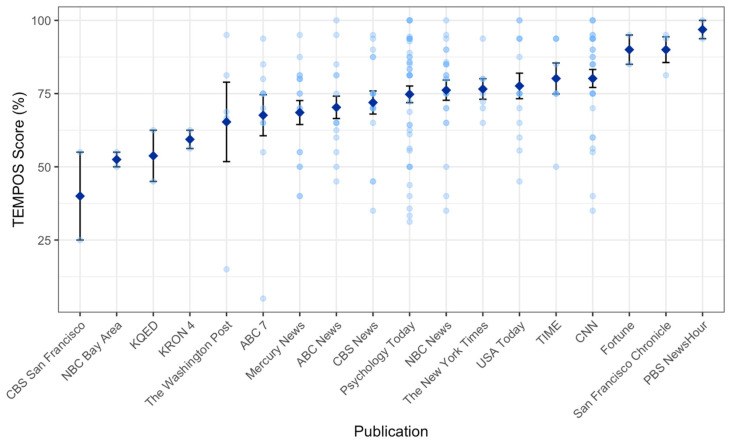
TEMPOS scores, sorted by publication. Each light blue point represents an article; dark blue diamonds indicate publication averages. Error bars represent standard error of the mean. Publications with less than 3 articles in the dataset were dropped from the plot.

We then examined how TEMPOS scores varied across and within publications (Figure 5). The average TEMPOS scores of each publication ranged from 4% to 96.9% (*M* = 76.6%, *SD* = 14.6%).

**Figure 6:**
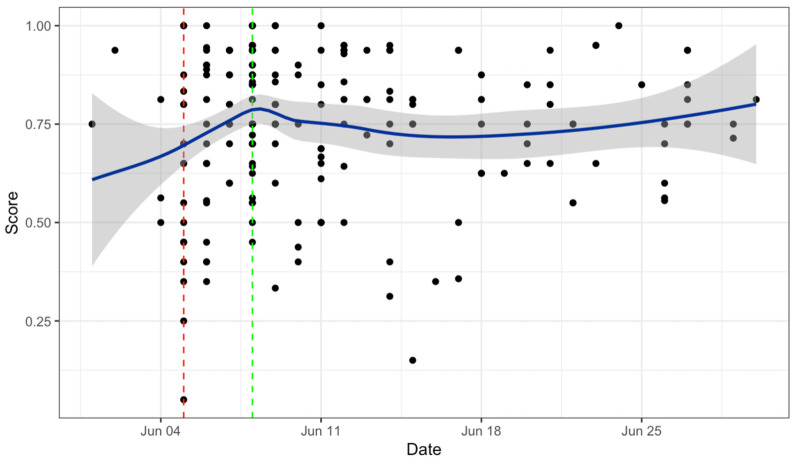
TEMPOS scores over time. Red and green dashed lines represent the deaths of Kate Spade and Anthony Bourdain, respectively.

Lastly, we examined whether overall adherence to the guidelines changed over the 1-month study period (Figure 6). An independent-samples t-test was conducted to compare TEMPOS scores on the day of Kate Spade’s death and the day of Anthony Bourdain’s death. Reporting on Bourdain’s death (*M* = 79.7%, *SD* = 13.9%) was significantly more adherent than reporting on Spade’s death (*M* = 62.3%, *SD* = 26.3%); *t*(23) = −2.82, *p* = 0.01.

## 4. Discussion

This paper describes the development and application of a novel, user-friendly, non-binary rating system to assess media adherence to suicide reporting recommendations. While many studies have examined the relationship between adherence to reporting recommendations and suicide rates, disparate measurement approaches make it difficult to draw meaningful comparisons across studies. To address these gaps, the current study explains the scale development process for the Tool for Evaluating Media Portrayals of Suicide (TEMPOS), as well as its application to a dataset of 220 media reports collected during a surge of suicide-related coverage. Results demonstrate the scale’s reliability, validity, and its utility as a tool for researchers, journalists, and public health professionals engaged in suicide prevention.

The application of TEMPOS yielded high inter-rater agreement among coders. Consistent with prior research [32,34], rater agreement levels were directly related to the degree of subjective interpretation required by raters. More concrete criteria (i.e., details about the suicide method) displayed higher levels of inter-rater reliability than more abstract criteria (i.e., glamorization of suicide), which may require more subjective judgment from the raters.

The application of TEMPOS to a dataset of suicide-related media articles from June 2018 demonstrated that adherence increased slightly following the death of Kate Spade, which aligns with previous findings that media reporting on Bourdain’s death was more guideline-adherent [38]. At the time of these highly publicized deaths, many media outlets received public criticism for the inappropriate ways that they reported on Spade’s death [38], which may have alerted media outlets to the importance of adhering to the reporting recommendations. TEMPOS provides a method for monitoring trends in reporting adherence over time, which may help shed light on the links among public suicides, media adherence levels, and suicide rates. This is a vital step in developing prevention strategies for combatting suicide contagion and protecting public health.

A critical contribution from this study is an illustration of how TEMPOS can be used by a range of constituents involved in the prevention of suicide. Professionals engaged in regional or broad-reaching prevention programs can use TEMPOS to better understand how media adherence to suicide reporting recommendations varies among criteria, across and within publications, and over time. TEMPOS can also be used to identify which reporting recommendations are commonly violated and which practices have already been widely adopted. For example, our analyses revealed that very few articles in the dataset featured suicide prevention and mental health resources, which aligns with previous research [29,30,38]. Systematic ratings from TEMPOS can yield powerful datapoints that allow suicide prevention professionals to develop more targeted and efficient interventions focused on improving adherence to the most commonly ignored recommendations, or media outlets in particular need of further training. TEMPOS can also be directly used by editors and publication leadership to determine if there are specific sections or reporters within their organizations that are in need of training. Importantly, TEMPOS also makes it possible to examine the impact of such trainings by providing a standardized way of measuring adherence before and after the training is administered. Taken together, the development, testing, and application of this novel rating scale for assessing adherence to suicide reporting recommendations provides a promising springboard for a diverse set of constituents to make strides in evidence-based suicide prevention efforts.

### Limitations & Future Directions

Several limitations should be noted. First, the dataset may not be fully representative of all news articles published in June 2018 because the article collection process relied on publicly accessible media. In addition, social media and articles shared via social media were not included in the dataset; however, social media is among the most common sources of news media for young people ages 18–29, and 48% of adults report receiving news from social media [39]. In addition to sharing and consuming news media, many people use social media to express personal experiences with mental health and suicide, which can further contribute to the spread of suicidal behavior [40]. Consequently, adapting TEMPOS to be suitable for assessing social media content is an especially important direction for future work. One promising example is the #Chatsafe Project in Australia, which introduced a set of evidence-based guidelines aimed at helping young people communicate about suicide safely online [41]. Future work can draw upon the #Chatsafe project and other research on the relationship between social media and suicide contagion to adapt TEMPOS for use with social media content.

Second, applying TEMPOS to every type of news article about suicide proved difficult. While the scale was easily applied to reports on individual suicide deaths, assessing articles that addressed suicide more broadly (e.g., articles discussing suicide trends, the general topic of suicide, or other related issues) was more challenging. Although efforts were made to expand the functionality of the tool (e.g., adding a “not applicable” option for some criteria), further work is needed to optimize the scale for application across a wide range of media types.

Third, under the current scoring system, all criteria are considered equal in significance when calculating a total score. While all ten criteria are important aspects of responsible reporting, failure to adhere to certain guidelines may result in higher risk for imitative behavior than others. For example, sharing specific details of the suicide method and location may be more likely to elicit copycat suicide attempts than the use of stigmatizing language [18]. Further development of the rating scale might consider assigning weights to criteria based on specific elements of reporting that have been identified as being most harmful. Furthermore, elements such as article size, tone, and ratio of positive to negative content are potentially important factors underlying the risk of imitative behavior [20,32,34]. Future iterations of TEMPOS may choose to leverage text analysis software programs such as Linguistic Inquiry & Word Count (LIWC) [42] to automatically calculate such variables and factor them into the overall TEMPOS score.

Finally, although TEMPOS offers a relatively efficient approach to measuring adherence, newsrooms often operate on extremely tight timelines. A primary aim of this work was to create a rating system that was complex enough to capture more nuance than a binary rating system, yet still accessible enough to be used by media professionals who are unfamiliar with the topic of suicide contagion. Ideally, media professionals would be able to use TEMPOS as a ‘self-check’ tool to assess the adherence levels of their content prior to publishing, and thereby reduce the risk of releasing harmful content. However, applying the scale prior to publication may not be realistic or feasible in the context of breaking news stories (e.g., the suicide of a high-profile figure). One way to further increase the practical utility of TEMPOS would be the use of artificial intelligence to automate evaluation of adherence to the reporting recommendations. Recent advances in machine learning and natural language processing may present an opportunity for TEMPOS to become partially or fully automated, which would allow for a wider range of applications. TEMPOS could also be used to intervene “upstream” by increasing reporters’ awareness of the reporting recommendations prior to scenarios in which they would need to apply them. For example, schools of journalism and publication companies could incorporate TEMPOS into their curricula, standards of practice, and professional training.

## 5. Conclusions

The application of the Tool for Evaluating Media Portrayals of Suicide (TEMPOS) has the potential to dramatically change how suicide is discussed and ultimately perceived. TEMPOS is a novel, user-friendly, and reliable tool for assessing adherence to suicide reporting recommendations that can be used by researchers, suicide prevention professionals, and media professionals alike. A key strength of TEMPOS is that it acknowledges the nuances in communication around suicide and the complexity of suicidal behaviors. In a departure from other rating scales, which typically employ binary measures of adherence, TEMPOS’s three-point scale allows raters to capture more nuance in adherence to reporting recommendations. As illustrated in this study, TEMPOS makes it possible to examine how media adherence to suicide reporting recommendations varies among criteria, across and within publications, and over time. Suicide prevention professionals seeking to work with media outlets to increase adherence can use TEMPOS to develop more targeted programming, both preemptively through education and training and during more urgent periods, such as during heightened-risk periods following surges of suicide-related coverage. Internally, media organizations can employ TEMPOS as an ongoing tool for self-assessment and monitoring. Ultimately, TEMPOS provides a platform for promoting widespread awareness of how reporting on suicide impacts individuals and communities, potentially leading to reduced stigma and improved visibility of a looming public health issue that is not commonly discussed.

## Figures and Tables

**Table 1:** Overview of TEMPOS Criteria.

#	Criterion Name
1	Framing of Suicide
2	Factual & Non-Speculative Information
3	Appropriate/Non-Stigmatizing Language
4	Details About Suicide Method/Scene
5	Details About Suicide Note
6	Visual Content
7	Reasons & Risk Factors for Suicide
8	Sensational Language
9	Glamorization of Suicide
10	Suicide Prevention/Mental Health Resources

Note: The full TEMPOS rating scale has been included as a Supplementary Material.

**Table 2:** Article Characteristics.

Characteristic		n (%)
Section		
	News	89 (40.5%)
	Blog	46 (20.9%)
	Health	28 (12.7%)
	Entertainment	24 (10.9%)
	Other	24 (10.9%)
	Opinion	5 (2.27%)
	Sports	4 (1.82%)
Region		
	National (USA)	178 (80.9%)
	Local (SF Bay Area)	42 (19.1%)
Publication Type		
	Broadcast News Company	115 (52.3%)
	Magazine	60 (27.3%)
	Newspaper	51 (23.2%)

**Table 3:** Pure Agreement and Weighted Kappa Values by Criterion.

Criterion	% Pure Agreement	κ (Linear Weights)
Framing of Suicide	73.64%	0.513
Factual & Non-Speculative Information	68.18%	0.452
Appropriate/Non-Stigmatizing Language	83.10%	0.587
Details About Suicide Method/Scene	91.82%	0.941
Details About Suicide Note	97.27%	0.975
Visual Content	92.27%	0.832
Reasons & Risk Factors for Suicide	76.82%	0.569
Sensational Language	73.18%	0.378
Glamorization of Suicide	75.90%	0.173
Suicide Prevention/Mental Health Resources	80.90%	0.743
AVERAGE	81.31%	0.616

## Data Availability

The dataset of articles and TEMPOS ratings can be provided upon request.

## Supplementary Materials

The following are available online at https://www.mdpi.com/article/10.3390/ijerph19052994/s1: The Tool for Evaluating Media Portrayals of Suicide, full rating scale.
