# Supplementary material for: The Tool for Evaluating Media Portrayals of Suicide (TEMPOS): Development and Application of a Novel Rating Scale to Reduce Suicide Contagion
Source: Int J Environ Res Public Health. 2022 Mar 4;19(5):2994. doi: 10.3390/ijerph19052994 (PMC8910134; doi:10.3390/ijerph19052994)
Supplement: Supplementary file 1 [file ijerph-19-02994-s001.zip › ijerph-1597021-supplementary.pdf]

T E M P O S

Tool for Evaluating Media Portrayals of Suicide

TEMPOS is the first tool that allows media professionals, public health officials, researchers, and suicide prevention experts to assess adherence to the recommended reporting guidelines with a user-friendly, standardized rating scale. The scale can be used to monitor changes in reporting over time and how reporting varies across articles, authors, and publications.

TEMPOS Criteria

1. How does the report frame the suicide?
2. Does the report include factual and non-speculative information about suicide?
3. Does the report use appropriate/non-stigmatizing language?
4. How does the report describe the suicide method and scene?
5. How does the report describe the suicide note?
6. What visual content does the report include?
7. How does the report describe risk factors and reasons for suicide?
8. Does the report use sensational language?
9. Does the report glamorize suicide?
10. Does the report include suicide prevention and mental health resources?

For each of the 10 criteria on the TEMPOS scale, the report can be scored as follows:

2 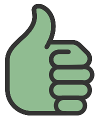 **HELPFUL**

- Helpful messaging
- Full adherence to the guideline

1 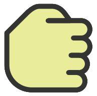 **MIXED**

- Mixed messaging
- Partial adherence to the guideline

0 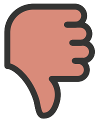 **HARMFUL**

- Harmful messaging
- Non-adherence to the guideline

TEMPOS was developed through a collaboration with the County of Santa Clara Behavioral Health Services Department's Suicide Prevention Program and the Stanford Department of Psychiatry and Behavioral Sciences' Media and Mental Health Initiative.

1. How does the report frame the suicide?

	DEFINITION	EXAMPLES
2 HELPFUL	Frames suicide as a preventable mental health outcome; reports that coping skills, support and treatment work for most people (recovery is possible).	"If you or someone you know is struggling with suicidal thoughts, help is available. With proper treatment and support, it is possible to get help for suicidal thoughts and other mental health challenges."
1 MIXED	Doesn't portray suicide as an escape or inevitable response to hardship, but fails to include that suicide is preventable and that resources are available to those who are struggling; may include a mix of these two portrayals.	
0 HARMFUL	Explicitly presents or strongly implies that suicide is a common, acceptable, or inevitable response to hardship; frames suicide as "a way out" or a way of taking control of one's circumstances.	"The young girl was being bullied at school and felt she had no other escape but to take her own life."<br>"In an official statement, Avicii's family stated that '[Avicii] really struggled with thoughts about Meaning, Life, Happiness. He could not go on any longer. He wanted to find peace.'"

2. Does the report include factual and non-speculative information about suicide?

	DEFINITION	EXAMPLES
2 HELPFUL	Includes information that is clearly factual in nature, not speculative. May include quotes or objective information from informed sources (e.g. people or organizations with mental health or suicide prevention expertise, and/or people with lived experience).	"Anne Shuchat M.D. is principal director at CDC. She told reporters, 'We did see increases in younger and older people. Essentially every age group other than those over 75.'"
1 MIXED	Does not include speculation/non-factual information, but also fails to provide factual information about suicide/mental health; may include a mix of these two portrayals.	
0 HARMFUL	Includes information that is clearly speculative (e.g., non-factual) about the causes of/reasons for suicide. Sources of information (e.g. friends/family/professionals) are not factually informed or are inaccurate/inappropriate.	"According to a friend, '[Avicii was] an artist who seemed to be having a slow-motion nervous breakdown brought on by the relentless pressures of success and a brutal touring schedule.'"<br>Headline: "A long-depressed Kate Spade was fixated on Robin Williams' suicide, sister claims."

3. Does the report use appropriate/non-stigmatizing language?

	DEFINITION	EXAMPLES
2 HELPFUL	Uses appropriate/non-stigmatizing language that is more neutral and treats suicide similarly to other causes of death (e.g. “died by suicide”).	“Anthony Bourdain died by suicide.”
1 MIXED	Uses a mix of inappropriate and appropriate language.	
0 HARMFUL	Uses inappropriate/stigmatizing language that implies criminality (e.g. “committed”), judgment, or positive connotations (e.g. “successful attempt”).	“Anthony Bourdain committed suicide.”

4. How does the report describe the suicide method and scene?

	DEFINITION	EXAMPLES
2 HELPFUL	Reports the death as a suicide but keeps information general and does not mention method.	“Famed fashion designer Kate Spade was found dead in an apparent suicide this past week.”
1 MIXED	Briefly mentions suicide method (e.g. asphyxiation, overdose) but does not include explicit details about the method used or the scene of the death.	
0 HARMFUL	Describes or depicts, in a detailed manner, the method and/or location of the suicide; ‘sets the scene’ by including information about what was found at the scene of the death, how the person was found, or the type of object used.	“Kate Spade, a fashion designer known for her sleek handbags, was found hanged in the bedroom of her Park Avenue apartment Tuesday in an apparent suicide, law enforcement officials said. The 55-year-old was found by housekeeping at about 10:20 a.m. Her husband and business partner Andy Spade was in the house at the time. The couple’s 13-year-old daughter was at school at the time and officials said a note was found at the scene telling her it was not her fault.”<br>“Kate Spade was found hanging from her doorknob with a red scarf.”

N/A Article is not about a specific person's suicide.

5. How does the report describe the suicide note?

	DEFINITION	EXAMPLES
2 HELPFUL	Does not mention a note or its contents; states that no note was found.	"No note was found."
1 MIXED	Reports that a note was found but does not include any content from the note.	
0 HARMFUL	Shares specific content drawn directly from a suicide note.	"Kate Spade left a heartbreaking note to her daughter in which she wrote, 'Bea – I have always loved you. This is not your fault. Ask Daddy!.'"<br>"Kate Spade left a note to her daughter telling her it wasn't her fault."
N/A	Article is not about a specific person's suicide.	

6. What visual content does the report include?

	DEFINITION	EXAMPLES
2 HELPFUL	Uses visual content of the person who died from school/work/ family or photo unrelated to suicide.	Visual content of decedent unrelated to suicide; stock image that is not related to suicide or death.
1 MIXED	Includes visual content of grieving/sad individuals, memorials, or funerals.	
0 HARMFUL	Includes any visual content (e.g. photos/videos) of the location or method of death.	Visual content of the scene or method of death, e.g. train tracks, scene with police tape, bridge, gun, noose, pills.
N/A	Absence of visual content.	

7. How does the report describe risk factors and reasons for suicide?

Score	Definition	Examples
2 HELPFUL	Acknowledges (in the body of the article or in a sidebar) the complexity of suicide and describes risk factors (e.g. mental illness, economic hardship, family issues) that give suicide context.	"Robin Williams struggled with depression for many years prior to his death. However, the causes of suicide are complex and often involve multiple factors, such as mental illness, relationship issues, economic hardship, substance use issues, bullying, or a recent loss. You can learn more about risk factors and warning signs at cdc.gov/suicide/factors."
1 MIXED	Does not speculate about reasons for death but does not include information about risk factors.	
0 HARMFUL	Oversimplifies or speculates on the reason for suicide; simplifies the issue of suicide by directly or indirectly attributing the death to a single reason or saying that it happened 'without warning'.	"Kate Spade kills herself after husband asks for divorce."<br>"The academic pressure at the school is so intense that it has driven multiple students to take their own lives."

8. Does the report use sensational language?

Score	Definition	Examples
2 HELPFUL	Reports on the death using non-sensational facts and language that focuses on the person's life rather than death (similar to reporting on non-suicide deaths); when referring to suicide rates, references best available data and uses words like 'increase' or 'rise'.	"Anthony Bourdain, celebrity chef, dead at 61."<br>"A recent CDC report shows that suicide rates have been rising steadily in the United States."
1 MIXED	Includes some sensitive language that focuses on the person's life rather than death, but also includes a few instances of sensational language/details about the death.	
0 HARMFUL	Includes shocking or provocative language/details about suicide designed to elicit an emotional response; uses sensational language (e.g. 'epidemic', 'skyrocketing', 'spike') when describing suicide rates.	"Devastating suicide cluster strikes local high school."<br>"Avicii: Death by broken glass..."<br>"Suicide rates have skyrocketed in recent years."

9. Does the report glamorize suicide?

Score		DEFINITION	EXAMPLES
2	HELPFUL	Does not portray suicide in a positive or glamorous manner; focuses on the life they lived rather than their death, acknowledges positive aspects of their life, as well as their struggles.	"Through the simple act of sharing meals, Bourdain showcased both the extraordinary diversity of cultures and cuisines, yet how much we all have in common – he was open about his mental health challenges and struggles with addiction throughout his life, and often discussed his struggles in writing and on his show, *Anthony Bourdain: Parts Unknown*. Tragically, Bourdain took his own life on Friday."
1	MIXED	Includes some content that glamorizes/portrays suicide in a positive light; portrays their life in an idealized or glamorized way without acknowledging struggles.	
0	HARMFUL	Majority of article content glamorizes suicide; includes several tributes or portrays suicide in a positive manner (e.g. ties suicide to heroism, romance, honor).	"It's telling that [Bourdain] killed himself in this picturesque, story tale village."<br>"Bourdain's shocking death by suicide led to an outpouring of grief and support from celebrities around the world."

10. Does the report include suicide prevention and mental health resources?

Score		DEFINITION	EXAMPLES
2	HELPFUL	Includes a suicide hotline/crisis number as well as additional resources for mental health care and suicide prevention, for example local mental health resources, suicide prevention organizations, websites.	You can call the National Suicide Prevention Lifeline at 1-800-273-TALK (8255) or text HOME to the Crisis Text Line at 741741.<br>***Additional Resources:***<br>American Foundation of Suicide Prevention (afsp.org)<br>American Association of Suicidology (suicidology.org)<br>National Institute of Mental Health (nimh.nih.gov)<br>Suicide Prevention Resource Center (sprc.org)
1	MIXED	Includes a hotline/crisis number, but does not provide any additional mental health resources/information.	
0	HARMFUL	Does not include any resources.	
